# Baetidae (Insecta: Ephemeroptera) of Aurès Mountains (Algeria): A New Species of the *Baetis alpinus* Species Group, with Notes on *Baetis* Laech, 1815 Biogeography within Maghreb

**DOI:** 10.3390/insects14110899

**Published:** 2023-11-20

**Authors:** Besma M. Dambri, Roman J. Godunko, Nadhira Benhadji

**Affiliations:** 1 Department of Ecology and Environment, Faculty of Natural and Life Sciences, University of Batna 2, Fesdis 05078, Batna, Algeria; besma.dambri@univ-batna2.dz; 2 Biology Centre of the Czech Academy of Sciences, Institute of Entomology, Branišovská 31, 37005 České Budějovice, Czech Republic; godunko@seznam.cz; 3 Department of Invertebrate Zoology and Hydrobiology, Faculty of Biology and Environmental Protection, University of Lodz, Banacha 12/16, 90237 Lodz, Poland; 4 State Museum of Natural History, National Academy of Sciences of Ukraine, Teatralna 18, 79008 Lviv, Ukraine; 5 Institute of Technology and Life Sciences–National Research Institute, Falenty, Hrabska Avenue 3, 05090 Raszyn, Poland

**Keywords:** Baetinae, mayflies, taxonomy, *Baetis* (*Baetis*) *dihyae* **sp. nov.**, new characters, COI, distribution, North Africa

## Abstract

**Simple Summary:**

The riverine ecosystem of the Aurès Mountains in Algeria is situated in the southern part of the Mediterranean basin. This unique region is an important centre of speciation, with a distinct number of regional endemics—some of which are still undescribed or very poorly studied. Given the importance of biomonitoring the ecological status of surface waters in this region, as well as the need to study and protect the diversity of aquatic fauna, information on the taxonomic composition and status of regional species is essential. Our attention was focused on the mayflies of the genus *Baetis* Leach, 1815 (Ephemeroptera: Baetidae), a typical element of the river macrozoobenthos within the Aurès Mountains. A new species of *Baetis* (*Baetis*) *dih*y*ae* **sp. nov.** is described for larvae and attributed to the *Baetis alpinus* species group using morphological and molecular evidence. The affinities of *Baetis* (*Baetis*) *dih*y*ae* **sp. nov.** to the closely related Palearctic species *Baetis* (*B.*) *alpinus* (Pictet, 1843); the western Alps endemic *Baetis* (*B.*) *nubecularis* Eaton, 1898; and *B.* (*B.*) *pasquetorum* Righetti & Thomas, 2002 (reported only from a few localities in southern France) are discussed in detail. The record and further description of a new species is the first contribution to the knowledge of the relatively diverse mayfly family Baetidae, distributed in the Aurès Mountains. Establishing a representative level of diversity of the macroinvertebrate fauna of the region is closely linked to both field studies and analyses of the main pathways of invasion of its individual elements. Using an extensive data set on the geology and historical biogeography of different faunal groups as well as analysing the diversity of the *Baetis alpinus* species-group representatives in Western Europe and the Maghreb, we examined in this contribution its colonisation pathways in North Africa.

**Abstract:**

A new species, *Baetis* (*Baetis*) *dihyae* **sp. nov.**, belonging to the *Baetis alpinus* species group, is described and illustrated based on larval material collected in the Aurès Mountains (northeastern Algeria) in 2020–2021. This new species is closely related to three European species, e.g., *Baetis* (*B.*) *alpinus* (Pictet, 1843); *B.* (*B.*) *nubecularis* Eaton, 1898; and *B.* (*B.*) *pasquetorum* Righetti & Thomas, 2002 by the combination of the following characteristics: (**i**) more than one short, stout bristle at the tip of segment II of the maxillary palp and (**ii**) a well-developed paracercus. However, the new species clearly differs from all congeners of the *Baetis alpinus* species group primarily by the (**a**) structure of mouthparts—with 14–18 long submarginal setae arranged in a single irregular row on the dorsal surface of the labrum; 2–6 short, stout bristles at the tip of segment II of the maxillary palp; and segment II of the labial palp without a considerably developed apico-internal lobe); (**b**) setation of abdominal terga, with a few triangular-shaped scales sparsely scattered near the posterior margin only; and (**c**) a well-developed paracercus, comprised of more than 50 segments. Primary data on the biology and distribution of this new species are provided, and molecular affinities are verified by the analysis of COI (barcode) sequences. Detailed notes on the distribution of mayfly species belonging to the *Baetis alpinus* species group common in Western Europe and the western part of North Africa are presented. The historical movement of *Baetis* representatives between Europe, North West Africa, and subsequently Algeria, with the land bridges ‘Strait of Gibraltar’ and ‘Strait of Sicily’ as colonization routes, is discussed in detail and identified in the present study as the Western Algeria colonization path and Eastern Algeria colonization path, respectively.

## 1. Introduction

*Baetis* Leach, 1815 is the most diverse genus of Order Ephemeroptera in the West Palaearctic [1]. Müller-Liebenau [2] arranged European representatives of this genus into eleven species groups supported later by Jacob [3]. One such species group is the Holarctic *Baetis alpinus* group which is closely related to the *B. pavidus* Grandi, 1949 and *B. lutheri* species groups [4].

The *Baetis alpinus* species group is morphologically distinct, inter alia by a dorso-ventrally flattened larva, with a shortened abdomen bearing dark spots and reduced paracercus (less than two-thirds of the cerci), reflecting the rheophilic character of this group. Moreover, the tarsal claws possess a pair of fine bristles subapically as defined by Bauernfeind and Soldán [4]. Recently, Godunko et al. [5] reported the distinguishing characters for several European species of the *Baetis alpinus* group and proposed a new set of features useful for the species delimitation. 

In the West Palearctic, *Baetis alpinus* (Pictet, 1843) is the most widespread group representative within the European mountain ranges [4], where it presents two lineages that radiate from the Alps to the Carpathians and to the Apennines and the Iberic peninsula [6,7]. On the other hand, in the Mediterranean islands, the occurrence of *B. alpinus* endemics is confirmed, and several are described, e.g., *B. cyrneus* Thomas & Gazagnes, 1984 from Corsica and *B. cypronyx* Godunko, Soldán & Staniczek, 2017 from Cyprus [5,8]. 

In northern Africa (namely in the Maghreb), populations of *B. maurus* Kimmins, 1938 heterospecific to the Iberian populations have been recorded from the Tafna river basin, whilst the species *B. berberus* Thomas, 1986, probably endemic to Morocco, was described from the High Atlas (Morocco), and *B. punicus* Thomas, Boumaїza & Soldán, 1983 was reported from the Maghreb area and a single locality in Spain [4,9]. There are also populations of some unspecified taxa attributed to “*Baetis* gr. *alpinus*” reported from Morocco [10] and the eastern part of Algeria [11,12], which have never been thoroughly taxonomically investigated. 

Because of growing interest, the North African region is currently experiencing a plethora of new Ephemeroptera species discoveries—of a distinctly endemic nature [10,11,12,13,14,15,16,17,18,19]. Hitherto, the most remarkable results have concerned the fauna of Algeria, the largest country of the Maghreb. In the Aurès region in northeastern Algeria, the first studies on the fauna of the Baetidae began at the same time as the first investigation of mayflies [20,21,22,23].

Due to lack of consistent investigation and the fragmentation of information about mayfly fauna of the Northern and Southern Aurès Mountains (including the Batna and Biskra Provinces) (see, e.g., Dambri et al. [19], Soldán and Thomas [24], Bebba et al. [25]), only several species from this area were reported in the comprehensive checklist of North Africa by Thomas [26]: with 14 reported mayfly taxa, 9 of them are representatives of the family Baetidae. Later, Bebba et al. [25] additionally reported *Choroterpes atlas* Soldán & Thomas, 1983 (Leptophlebiidae), and recently Dambri et al. [19] described a new species *Ecdyonurus aurasius* Dambri, Benhadji & Sartori, 2022 (Heptageniidae) from the Aurès Mountains occurring along with *B. chelif* Soldán, Godunko & Thomas, 2005 and *B. sinespinosus* Soldán & Thomas, 1983. 

In this contribution, we aim to broaden the knowledge of this spectacular species group within the Maghreb region on the occasion of the description of *Baetis* (*Baetis*) *dihyae* **sp. nov.** from several valleys in the Aurès Mountains (Algeria). Additional objectives of this contribution are to discuss the differential diagnosis of a new species from other closely related representatives of the *Baetis alpinus* species group based on both morphological and molecular data. Furthermore, we provide the first biogeographical analysis of this species group in the Maghreb region, with investigation of possible colonization pathways in the context of known paleoclimatic and geologic events.

## 2. Materials and Methods

### 2.1. Sampling, Type Series, Comparative Material

**Figure 1:**
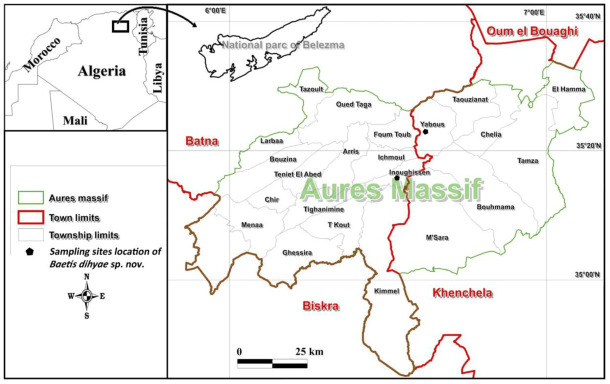
Map of sampling sites in the Aurès Mountains (Algeria).

The larval specimens of *B. dihyae* **sp. nov.** were collected with a water net between June 2020 and October 2021 in the Yabous and Inoughissen sites within the Aurès Mountains (Figure 1). All larvae were preserved in 95% ethanol and stored at 4 °C for long-term preservation. The type material of *B. dihyae* sp. nov is organised into the following collections: holotype (male larva), 1 paratype (1 female larva), and 4 paratypes (4 female larvae) mounted on slides using Faure liquid and 3 paratypes (3 male larvae) and 2 paratypes (1 female and 1 male larvae) used for SEM are in the collection of R.J. Godunko at the Institute of Entomology, Biology Centre of the Czech Academy of Sciences (IE-BC CAS); 1 paratype of a female larva is slide mounted and in the collection of N. Benhadji at the Institute of Technology and Life Sciences, National Research Institute, Szczecin branch, Poland (ITP-PIB); and 14 paratypes (5 male and 9 female larvae) are stored in the collection of B.M. Dambri at the Functional Ecology and Environmental Laboratory, University Batna 2, Algeria (UB2).

Type series of *Baetis* (*Baetis*) *dihyae* **sp. nov.**

**HOLOTYPE**: mature male larva, Algeria, Wilaya de Khenchela, wadi Ekehal, Yabous region, Yabous site, 35°21′11″ N, 6°38′35″ E, 1420 m. a.s.l., 23.II.2021, Dambri B.M. leg. [IE-BC CAS].

**Paratypes**: 1 mature female larva (the same date and site as holotype), 3 mature male larva (same site as holotype, 28.X.2021), 5 female and 1 male larvae (same site as holotype, 06.VII.2020), Dambri B.M. leg. (IE-BC CAS); 1 female larva mounted on slide, Algeria, Wilaya de Batna, wadi Abiod, Ichmoul region, Inoughissen site, 35°16′42″ N, 6°32′34″ E, 1670 m. a.s.l.; 1 female larva, ibid, 18.IV.2020, Dambri B.M. leg. (IE-BC CAS); 7 female larvae (same site as holotype, 06.VI.2021), Dambri B.M. leg.; 3 female and 1 male larvae, Algeria, Wilaya de Batna, wadi Abiod, Ichmoul region, Inoughissen site, 35°16′42″ N, 6°32′34″ E, 1670 m. a.s.l., 16.II.2020, Dambri B.M. leg. (IE-BC CAS).

Comparative material.

*Baetis alpinus* (Pictet 1843): 35 larvae, Ukraine, Zakarpats’ka region, Yasinia village, the mouth of Lazeshchyna river, 19–20.05.2006, Godunko R.J. leg. (IE CAS); 43 larvae, ibid, Bilyn village, W slope of the Shtev’era Mountain, small unnamed stream (left-bank tributary of the Chorna Tysa river), 19–20.05.2006, Godunko R.J. leg.; 22 larvae, ibid, E vicinity of Yasinia village, the bridge towards Chorna Tysa village, 26–27.06.2006, Godunko R.J. leg.; 57 larvae, ibid, Bilyn village, W slope of the Shtev’era Mountain, small unnamed stream (left-bank tributary of the Chorna Tysa river), 26–27.06.2006, Godunko R.J. leg.; 45 larvae, ibid, mouth of Keveliv stream, vicinity of Kvasy village, Keveliv settlement, 09–10.08.2006, Godunko R.J. leg. [all in the collection of the State Museum of Natural History, NAS Ukraine, Lviv].

*Baetis maurus* Kimmins, 1938: 2 male imagines and one larva, Spain, Sierra de Cazorla Arroyo de Liharejos, 22.06.1984, Soldán T. leg.; 7 larvae, ibid, Sierra de Cazorla, Rio Quadalquivir-mas abajo del Vadillo, 22.06.1984, Soldán T. leg. (all in the collection of the Institute of Entomology, IE-BC CAS).

*Baetis nubecularis* Eaton, 1898: 24 larvae, Switzerland, L’abbaye, Source de la Lionne, 1040 m a.s.l., 31.05.2006, Wagner A. det. & leg. (all in the collection of the Institute of Entomology, IE-BC CAS).

### 2.2. Morphological Study

Line drawings were made using a Zeiss Axioplan microscope with an Olympus BX41 camera lucida. Material was observed with the binocular microscope Leica M205 C. Photographs of larvae were taken using a Leica Z16 APO macroscope and processed with Leica Application Suite™ Version 3.1.8 to obtain combined photographs with enlarged depth of field. Photographs were subsequently enhanced with Adobe Photoshop™ CS3.

Two specimens used for SEM investigation were dissected and dehydrated through a stepwise immersion in ethanol and then dried by critical point drying and then mounted on SEM stubs and coated with gold by sputtering using Polaron PS 100. Observations were taken on the scanning electron microscope Jeol JSM 7401F at 4 kV in the Laboratory of Electron Microscopy, Institute of Parasitology, Biology Centre, CAS (České Budějovice, Czech Republic).

### 2.3. Terminology 

In this contribution, the terminology and corresponding acronyms used follow those proposed by Godunko et al. [27] (for the representatives of the subgenus *Rhodobaetis* Jacob, 2003); Godunko et al. [5] (for the species from *Baetis alpinus* species group); Godunko et al. [28] (for the species of the genus *Nigrobaetis* Novikova & Kluge, 1987) are used to describe the body setation (e.g., to characterize the types of stout setae and scales). Initially classification evolved based on the classification and terminology proposed by e.g., Gaino and Rebora [29,30] as a part of SEM studies of the representatives of the genus *Baetis* Leach, 1815, where several acronyms were proposed (e.g., *FT* for designation of flat-tipped sensillum, *B* for sensillum basiconicum, and *Hr* for hair-like setae).

### 2.4. Molecular Analysis

Three specimens *B. dihyae* **sp. nov.** have been successfully barcoded (Table 1). DNA extractions are conserved in the Institute of Biology of the University of Szczecin, Poland. We isolated DNA using DNA Isolation Kit: GeneMATRIX Tissue DNA Purification Kit (EURX) (cat. no. E3550); for PCR, we used PCR Polyerase: DreamTaq™ Hot Start Green PCR Master Mix (Thermofischer Scientific, Waltham, MA, USA) (cat. no. K9021) to the mitochondrial cytochrome oxidase I gene (COI) 658 bp fragments with LCO1490 and HCO2198 primers [31]. PCR cycles were performed according to Benhadji et al. [9]. We verified PCR success by electrophoresis using the ladder MassRuler DNA Ladder Mix, ready-to-use (Thermofischer Scientific) (cat. no. SM0403), and finally, we purified PCR products using Kit: GeneMATRIX PCR/DNA Clean-Up Purification Kit (EURX) (cat. no. E3520). After purification, we sent samples for sequencing to Macrogen (5 uL of starter + 5 µL of DNA sample) (Address: Meibergdreef 57 1105 BA, Amsterdam, The Netherlands. website link: https://order.macrogen-europe.com/# (accessed on 28 January 2022). We edited, trimmed, and assembled forward and reverse sequence reads using Geneious 10.2.6 [32], with sequence alignments available in the Supplementary Materials: Alignment S1 Using Molecular Evolutionary Genetics Analysis Version 11 (MEGA11); we aligned sequences of each taxon with analogue genus or species selected from Genbank or BoldSystem database [33,34]. To perform the biomolecular reconstruction tree, we aligned our sequences with sequences from *B. alpinus* [6,35,36,37,38,39,40,41,42,43], *B. cyrneus* [41,42], *B. maurus* [9,38] haplogroups and *B. melanonyx* [6,42,43] as an outgroup. We also performed the reconstruction in MEGA11, selecting the Tamura–Nei model to produce our tree with neighbor-joining analysis, with 1000 normal bootstrap replicates. The GenBank accession numbers for the 3 new sequences (three specimens of a new species) used in the molecular study are given in Table 1; the nomenclature for the gene sequences follows Chakrabarty et al. [44].

## 3. Results

### 3.1. Taxonomy

*Baetis* (*Baetis*) *dihyae* Benhadji, Dambri & Godunko, **sp. nov.**
LSID urn:lsid:zoobank.org:act:C60D7A6B-4FA1-46B5-8E35-A538C2157C96
Figure 2, Figure 3, Figure 4, Figure 5, Figure 6, Figure 7, Figure 8, Figure 9, Figure 10, Figure 11, Figure 12, Table 2 and Table 3.

**Table 2:** Indexes and corresponding acronyms of larval setation of *Baetis* (*Baetis*) *dihyae* **sp. nov.** All types of scales and stout and tiny setae are illustrated in Figure 5, Figure 6, Figure 7, Figure 8, Figure 9, Figure 10, Figure 11 and Figure 12. Description of body setation is given according to Godunko et al. [5,27] and Godunko et al. [28].

Type of Larval Setation (Indexes)	*Baetis* (*Baetis*) *dihyae* sp. nov.	
	Acronyms	Measurements (µm)
*1. Stout setae* (*Sensillum chaeticum)* (*thick bristles set in a socket*, *emerge from the ventral surface of scape and pedicel and all over the body)*	*STS*	
1.1. Pointed apically	*STS-p*	
1.1.1. Elongated, with distinctly convergent margins, widest at the base	*STSe-p*	18.03–40.96
1.1.2. Middle, with distinctly convergent margins, widest at the base	*STSm-p*	12.91–17.00
1.1.3. Small, with distinctly convergent margins, widest at the base	*STSs-p*	11.60–16.98
1.2. Blunt or bluntly pointed apically	*STS-bp*	
1.2.1. Middle, with smoothly convergent margins, widest at the base or near to the base	*STSm-b*	14.00–21.80
1.3. Rounded apically	*STS-ov*	
1.3.1. Oval-shaped	*STS-ov*	
1.3.1.1. Middle, widest near to the middle of seta length	*STSm-ov*	11.06–11.72
1.3.1.2. Small, widest near to the middle of seta length	*STSs-ov*	6.37–11.00
*2. Tiny setae*	*FT*, *B*, *Hr*	
2.1. Flat-tipped sensillum (uniporousbasiconic sensilla with their apical flange adhering to the cuticle)	*FT*	8.18–10.60
2.2. Sensillum basiconicum (uniporousbasiconic sensilla without apical flange)	*B*	10.96–15.13
2.3. Short hair	*Hr1*	15.92–20.12
2.4. Long hair	*Hr2*	20.71–21.75
2.5. Sensilla campaniformia (a few setae along of the distal margin of the pedicel)	*Ca*	10.72–15.21
2.6. Long, thin hair-like setae (body surface)	*Hr*	75.34–161.17
2.7. Sensilla Trichodea (the most common sensilla in the nymph are sensilla trichodea single or in groups, located mostly along the distal border of each flagellar segment)	*TR*	7.79–13.56
*3. Scales*	*SC*	
3.1. Tongue-shaped scales (body surface, e.g., abdominal terga)	*SC-tg*	10.65–13.21

**Table 3:** Morphological characters of *Baetis* (*Baetis*) *dihyae* **sp. nov.** (Figure 5, Figure 6, Figure 7, Figure 8, Figure 9, Figure 10, Figure 11 and Figure 12) and closely related species; a set of diagnostic characters proposed by Godunko et al. [5] and tested for representatives of the *Baetis alpinus* species group. Important diagnostic characters are marked in grey. Quotient *q* represents the degree of asymmetry of labial palps, proposed by Sroka et al. [45]. Description of body setation is given according to Godunko et al. [5,27,28] and measured in Supplementary Materials Alignement S1. Other characters are analysed based on material housed in the collection of the Institute of Entomology, BC CAS (see Section 2.1), and given according to Müller-Liebenau [2], Jacob [3], Bauernfeind and Soldán [4], Thomas et al. [46], Thomas and Dia [47,48], Péru and Thomas [49], Kluge and Novikova [50], Sroka et al. [45], and. Important differences in characters are marked in grey.

No.	Characters	*Baetis dihyae* sp. nov.	*Baetis nubecularis* Eaton, 1898	*Baetis pasquetorum* Righetti & Thomas, 2002	*Baetis alpinus* (Pictet, 1843)
*Body*					
1.	Body length	9.50–10.20	6.00–7.00	4.50–7.50	6.00–10.50
*Head*					
2.	Setation of clypeus	solitary *B* and *Hr*, and occasional *FT*	solitary *B*, *Hr* and *FT*	solitary *B*, *Hr* and *FT*	solitary *B*, *Hr* and *FT*
3.	Setation of scape and pedicel	*FT* and *Hr*; more abundant *B* setae	*FT* and *Hr*; more abundant *B* setae	occasional *FT*, *B* and *Hr*	solitary *FT* and *Hr*; more abundant *B*
4.	Frontal suture	widely U-shaped	U-shaped	widely V-shaped	widely V-shaped
*Mouthparts*					
5.	Labrum: shape	moderately oblong-shaped, slightly trapezoidal	nearly oblong-shaped and rectangular	distinctly oblong-shaped, strongly rectangular	distinctly oblong-shaped, nearly rectangular
6.	Labrum: mean width/length ratio	1.40–1.45	1.40–1.60	2.10–2.15	1.50–1.75
7.	Labrum: number of long submarginal setae	1 + 14–18	1 + 12–16	1 + 13–18	1 + 18–22
8.	Labrum: number of long marginal setae	6–12	5–6	2–4	up to 10
9.	Labrum: lateral margins [posteriorly]	markedly convergent	slightly convergent	slightly convergent	slightly convergent
10.	Mandibles: outer mandibular incisor group	relatively broad and rounded at apex, moderately separated from inner incisor group	relatively broad, rounded to bluntly pointed at apex, moderately separated from inner incisor group	broadly rounded and truncate at the apex, partly fused with inner incisor group	relatively broad, truncate at apex, partly fused with inner incisor group
11.	Mandibles: number of teeth of inner incisor group	3–4	3	3–4	3–5
12.	Mandibles: number of teeth on prostheca	8–10	6–8	6–8	5–9
13.	Maxillary palps: number of stout setae at the tip of distal segment	2–6	1–5	1–4	5–14
14.	Paraglossae: number of regular rows of apical bristles	2	2	2	3
15.	Paraglossae: number of bristles on outer margin	6–10	3–7	?	6–8
16.	Paraglossae: number of setae on ventral surface	4−5	3–4	?	5–6
17.	Labial palp: apico-internal lobe of segment 2	small, not developed	small, not developed	moderately developed	moderately or well-developed
18.	Labial palps: shape of segment 3	nearly symmetrical and evenly rounded	nearly symmetrical, short and slender, evenly rounded	nearly symmetrical, relatively elongated and slender	nearly symmetrical and evenly rounded
19.	Labial palps: mean length/width ratio of segment 3	0.95–1.10	0.90–1.00	1.15–1.25	0.85–0.95
20.	Labial palps: number of stout setae on dorsal surface of segment 3 apically	18–25	14–20	12–18	18–28
21.	Labial palps: number of stout setae on dorsal surface of segments 2 and 3	6	6	5	5–7
22.	Labial palps: degree of asymmetry (quotient *q*)	1.00–1.08	1.20–1.33	1.00–1.08	0.95–1.05
*Thorax and legs*					
23.	Shape of sternal protuberances on meso- and metathorax	prominent, pointed	small, pointed	?	prominent, pointed
24.	Outer margin of femora: shape of long bristles	slender, bluntly pointed and obtuse apically	slender, pointed apically	slender, pointed apically	slender, pointed apically
25.	Outer margin of femora: number of rows of long bristles proximally and centrally	2–3	1–2	2–3	2–4
26.	Outer margin of femora: shape of submarginal stout setae	*STSs-bp*, *STSm-bp*, *STSs-p*, *STSm-p*	*STSs-bp*, *STSm-bp*	*STSs-bp*, *STSm-bp*	*STSs-bp*, *STSm-bp*, *STSs-p*, *STSm-p*
27.	Outer margin of tibia: shape of stout setae	*STSs-bp*, *STSm-bp*, *STSs-p*, *STSm-p*	*STSs-bp*, *STSm-bp*	*STSs-bp*, *STSm-bp*	*STSs-bp*, *STSm-bp*, *STSs-p*, *STSm-p*
28.	Tarsal claw: number of strong teeth	8–11	9–11	10–13	8–12
29.	Tarsal claw: number of rows of marginal teeth	one	one	one	one
30.	Tarsal claw: two subapical hair-like setae	present	present	present	present
*Abdomen*					
31.	Surface of terga: scales	present, not numerous	absent	absent	present
32.	Surface of terga: scales sockets	present, not numerous	absent	absent	present
33.	Surface of terga: shape of scales	*SC-tg*	absent	absent	*SC-tg*
34.	Posterior margin of terga II–VIII: shape of spines	broad triangular, nearly symmetrical, bluntly pointed or occasionally pointed apically	broad triangular, nearly symmetrical (occasionally obtuse), bluntly pointed or pointed apically	broad triangular, nearly symmetrical, bluntly pointed or pointed apically	broad triangular, nearly symmetrical (occasionally obtuse), bluntly pointed or pointed apically
35.	Posterior margin of terga III–VIII (IX): submarginal irregular row of smaller spines	present	absent	absent	absent
36.	Shape of gills I and VII	moderately asymmetrical	asymmetrical	asymmetrical	asymmetrical
37.	Shape of gills II−V	asymmetrical	asymmetrical	asymmetrical	asymmetrical
38.	Paraproct plate (inner margin): number of marginal spines	1	6–9	10–15	4–9
39.	Paraproct plate (inner margin): number of submarginal stout setae	–	1–3	1–3	2–4
40.	Paraproct plate (inner margin): shape of submarginal stout setae	*STSs-bp*, *STSm-bp*	*STSs-bp*, *STSm-bp*	*STSs-bp*, *STSm-bp*	*STSs-p*, *STSm-p*
41.	Paraproct plate (surface centrally): type of setation	*Hr*, *B*	*Hr*, *B*	*Hr*, *B*	*Hr*, *B*
42.	Paracercus	app. 1/2 of cerci length; 50–60 segments	1/3–1/2 of cerci length; 47–56 segments	1/3 of cerci length; 25–45 segments	1/2 of cerci length; 32–42 segments
43.	Cerci and paracercus: posterior margin of segments	row of broad triangular spines, alternating with *B*	row of broad triangular spines, alternating with *B*	row of broad triangular spines, alternating with *B*	row of broad triangular spines, alternating with *B*

**Figure 2:**
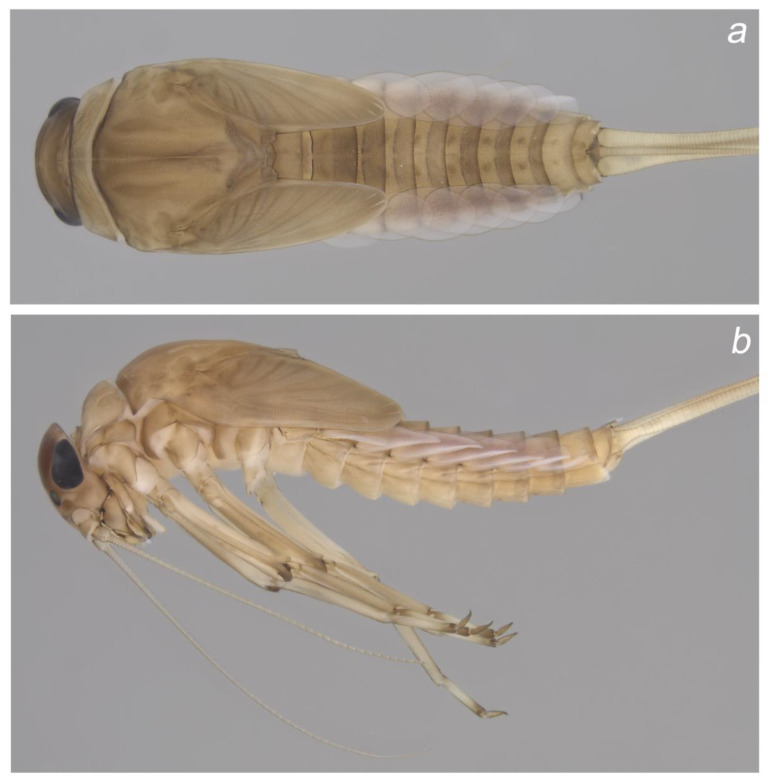
Colour pattern of *Baetis* (*Baetis*) *dihyae* **sp. nov.** larva, male, paratype (material from type locality): (**a**) body, dorsal view; (**b**) body, lateral view. Without scale bars.

**Figure 3:**
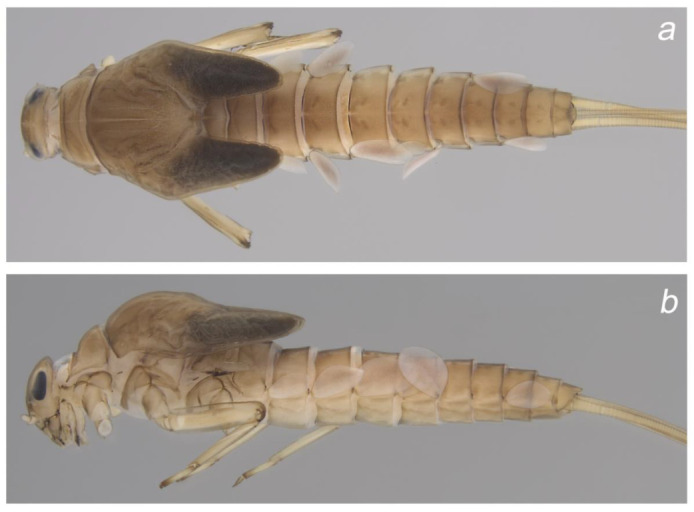
Colour pattern of *Baetis* (*Baetis*) *dihyae* **sp. nov.** larva, female, paratype (material from type locality): (**a**) body, ventral view; (**b**) body, dorsal view. Without scale bars.

**Figure 4:**
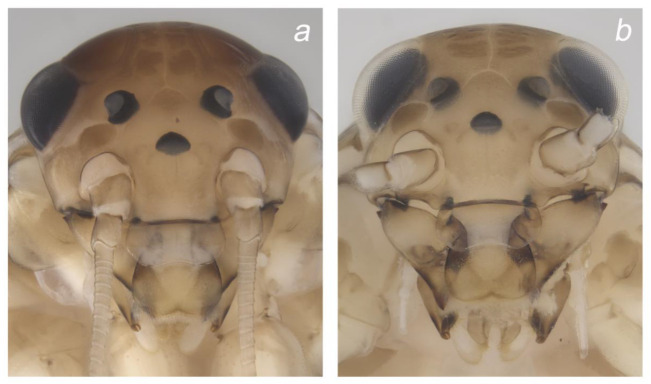
Colour pattern of *Baetis* (*Baetis*) *dihyae* **sp. nov.** larva (**a**,**b**) paratypes; material from type locality: 13 head, dorsal view: (**a**) male and (**b**) female. Without scale bars.

**Figure 5:**
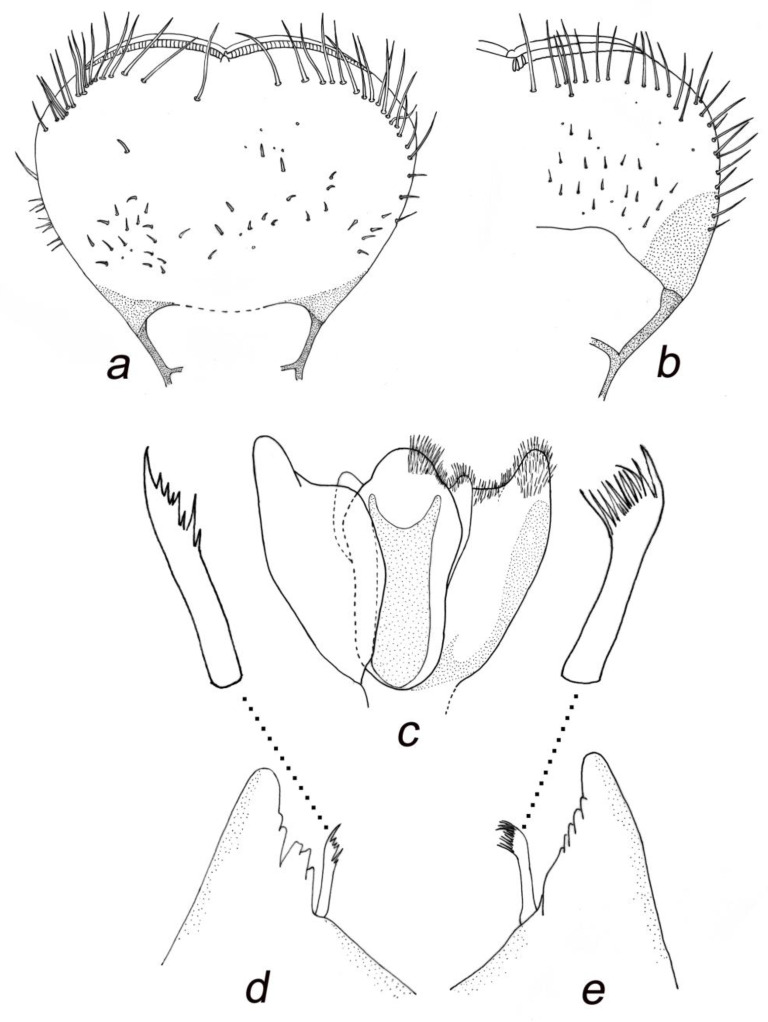
*Baetis* (*Baetis*) *dihyae* **sp. nov.** larva, paratypes, details of mouthparts: (**a**,**b**) shape of labrum, dorsal view; (**c**) hypopharynx (right half dorsal view, left half ventral view); (**d**) right mandible (incisors and prostheca), dorsal view; (**e**) left mandibular (incisors and prostheca), dorsal view. Without scale bars.

**Figure 6:**
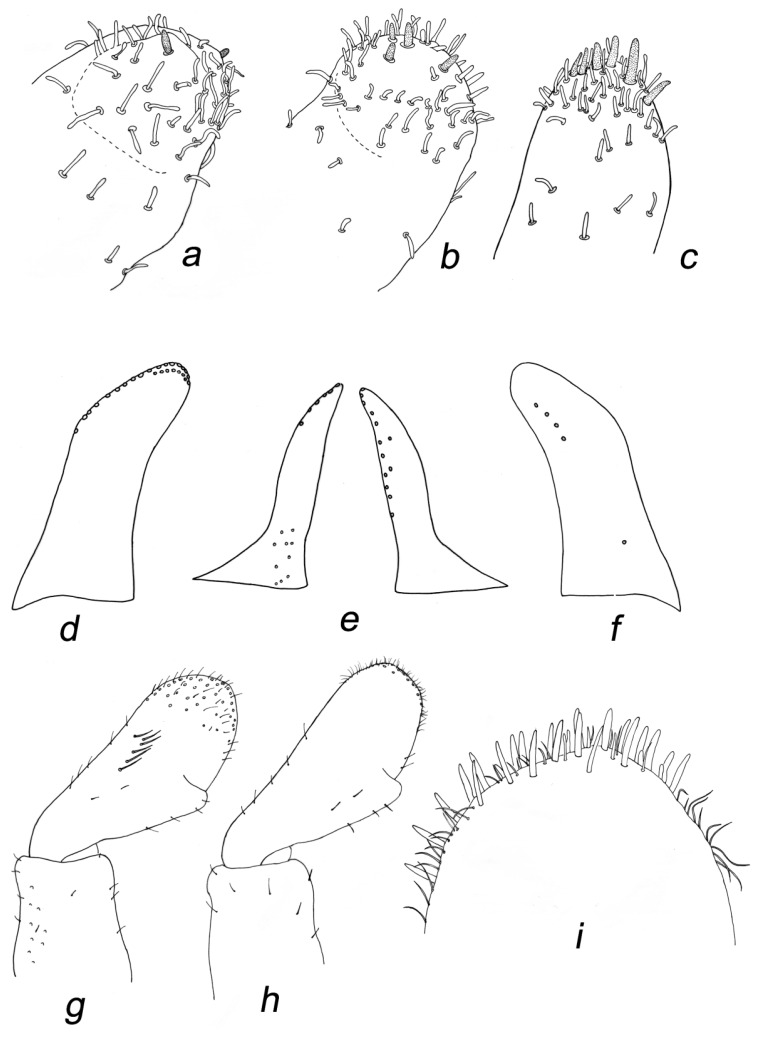
*Baetis* (*Baetis*) *dihyae* **sp. nov.** larva, paratypes, details of mouthparts: (**a**–**c**) apical part of maxillary palp, dorsal view; (**d**,**f**) paraglossa left and right, respectively, ventral view; (**e**) glossa; ventral view; (**g**,**h**) shape of third segment of labial palps, ventral view; (**i**) apical part of labial palp, ventral view. Without scale bars.

**Figure 7:**
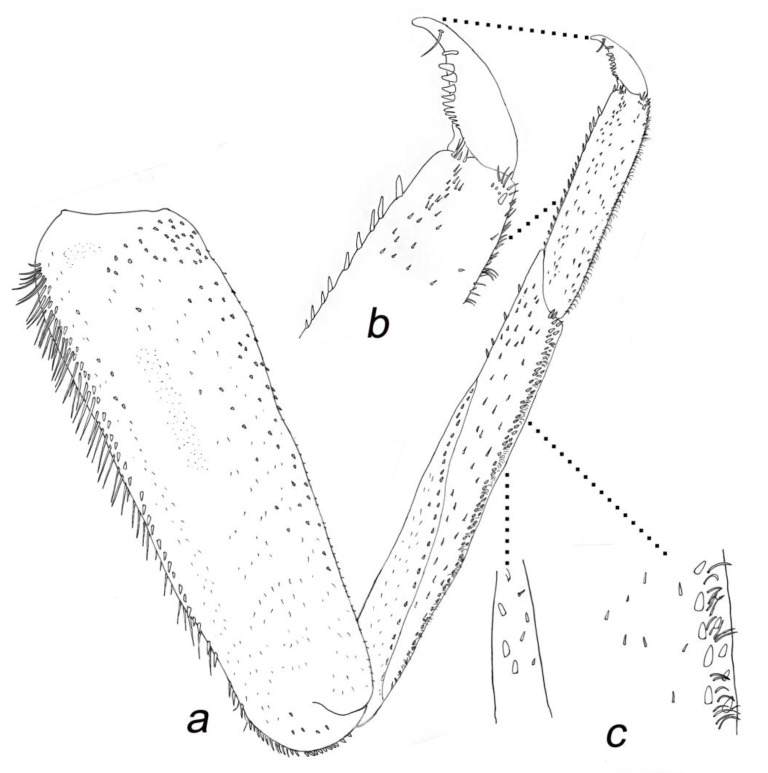
*Baetis* (*Baetis*) *dihyae* **sp. nov.** larva, paratype, hind leg: (**a**) general dorsal view; (**b**) tarsal claw, dorsal view; (**c**) outer margin of tibia, dorsal view. The sections (**b**,**c**) marked by dotted line. Without scale bars.

**Figure 8:**
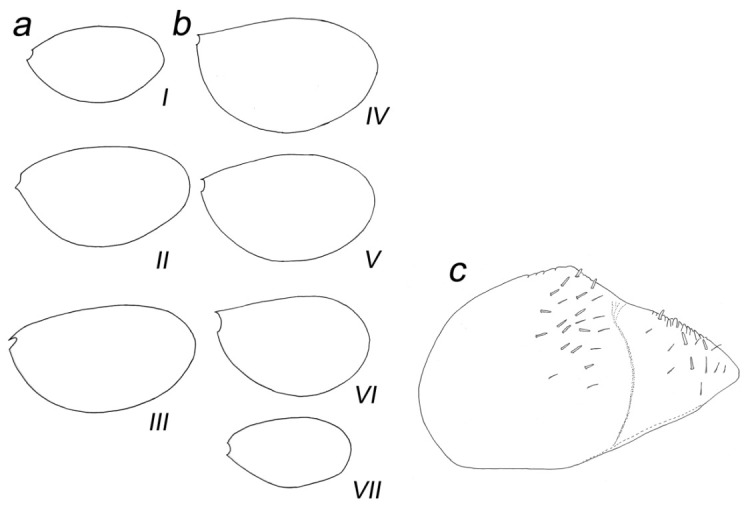
*Baetis* (*Baetis*) *dihyae* **sp. nov.** larva, paratype, gills: (**a**) gill I–III; (**b**) gill IV–VII; (**c**) details of paraproct, ventral view. Roman numbers refer to the respective gill pairs. Without scale bars.

**Figure 9:**
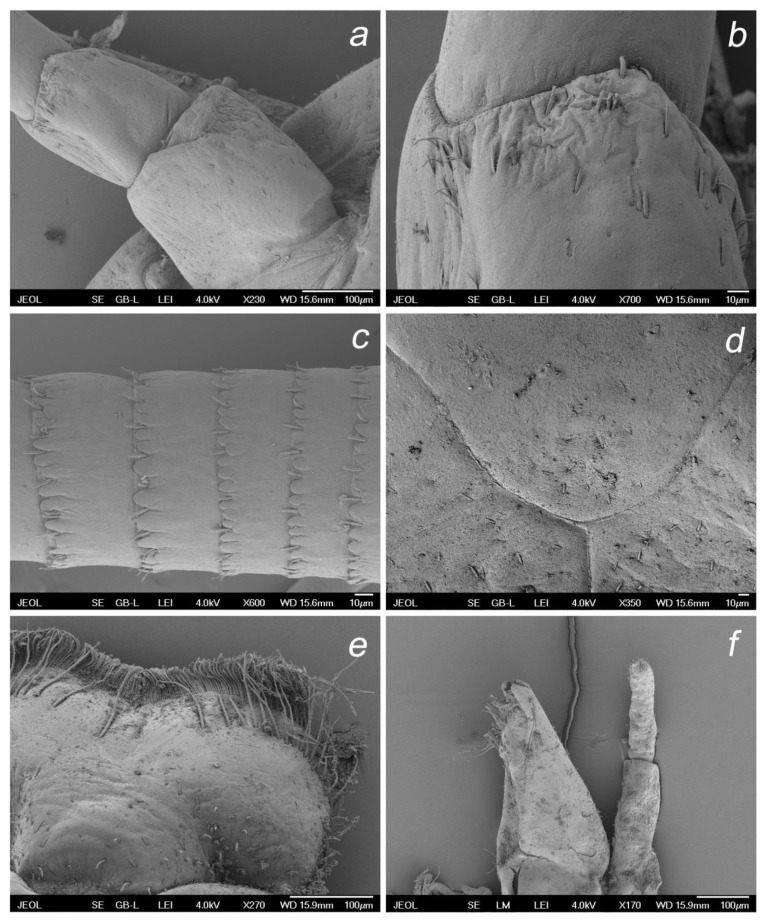
*Baetis* (*Baetis*) *dihyae* **sp. nov.** larva, SEM: (**a**) antenna (scapus and pedicellus); (**b**) details of pedicellus; (**c**) part of flagellum; (**d**) U-shaped sutures on head, dorsal view; (**e**) details of labrum bristles, dorsal view; (**f**) left maxilla and maxillary palp, ventral view.

**Figure 10:**
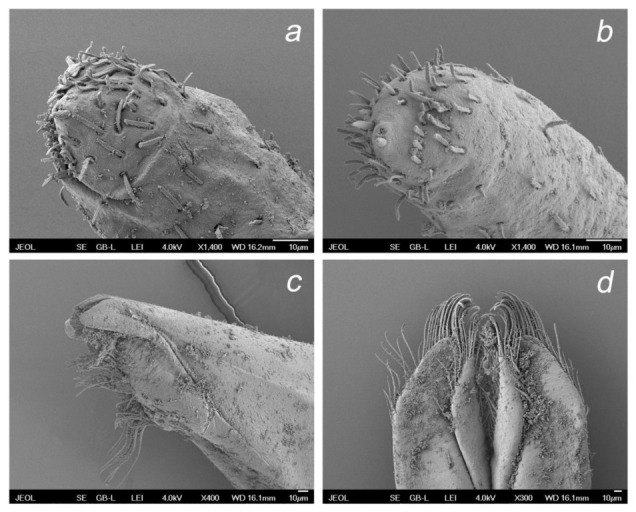
*Baetis* (*Baetis*) *dihyae* **sp. nov.** larva, SEM: (**a**) apical part of right maxillary palp; (**b**) apical part of left maxillary palp; (**c**) apical part of maxilla; (**d**) glossae and paraglossae, dorsal view.

**Figure 11:**
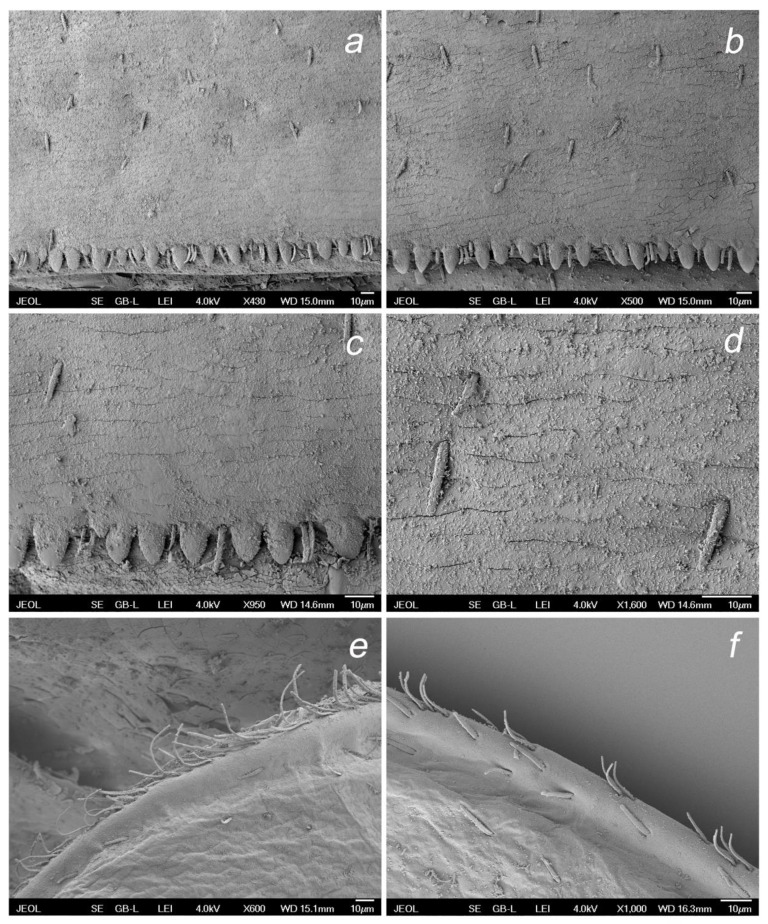
*Baetis* (*Baetis*) *dihyae* **sp. nov.** larva, SEM: (**a**) details of posterior margin and surface of terga III; (**b**) details of posterior margin and surface of terga VI, dorsal view; (**c**) details of posterior margin terga I, centrally; (**d**) details of surface of terga I, centrally; (**e**) outer margin of gill VI; (**f**) details of outer margin of gill VI.

**Figure 12:**
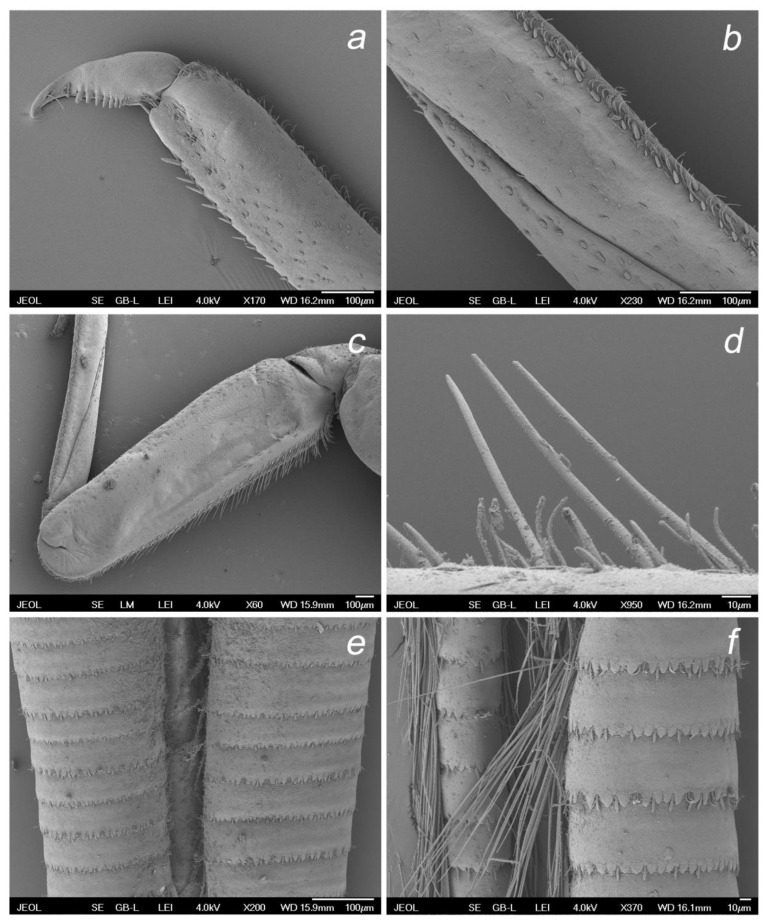
*Baetis* (*Baetis*) *dihyae* **sp. nov.** larva, SEM: (**a**) middle tarsus and tarsal claw, apically; (**b**) middle tibia, centrally; (**c**) middle femora, general view; (**d**) setae of outer margin of forefemora; (**e**,**f**) cerci and paracercus, general view.

**Etymology.** The species name “dihyae” is derived from the name of the Amazigh queen Dihya, a historical character who was known as a symbol of people who refuse to be subjugated and are willing to fight to retain their freedom. 

**Diagnosis** (based on larvae). The new species can be easily distinguished from all other representatives of the *Baetis alpinus* species group by the combination of following diagnostic characters: (**i**) head with frontal suture widely U-shaped; (**ii**) tip of segment II of the maxillary palp with up to six short stout setae; (**iii**) surface of terga: anterior margin with small groups of sparsely scattered tongue-shaped scales (*SC-tg*); (**iv**) submarginal irregular row of small spines on the posterior margin of terga III–VIII; (**v**) paraproct with occasional inconspicuous spine on anterior inner margin of plate; surface of the paraproct plate with occasional 1–3 stout submarginal setae of different size (*STSbp*); (**vi**) well-developed paracercus (more than 50 segments of paracercus comprise app. 1/2 of cerci length). 

Additionally, typical for *Baetis alpinus* species group, tongue-shaped scales [*SC-tg*] 10.65−13.20 µm in length were recognized (Table 2; see also Godunko et al. [5]). The body surface is covered also with medium to small stout setae [*STS*], as well as sensillae of several types (see Table 2).

**Description.** Mature larvae. Body length 9.50–10.20 mm (male larvae slightly smaller than female larvae), length of cerci 5.50–5.80 mm (app. 0.5× body length); paracercus well developed, length of paracercus 3.0–3.3 mm.

**Cuticular coloration** (Figure 2, Figure 3 and Figure 4). General colour yellowish grey to dirty yellow; head and thorax dark yellowish-brown to brown. Head light brown; genae and clypeus yellow to yellowish-brown; frons and vertex darker light brown to brown (Figure 4a,b). Two marked brown spots between base of antenna, ocellus, and eye. Facetted surface of larval turbinate eyes brown. Antennae light brown, flagellum paler than scape and pedicel. 

Pronotum dirty yellow to brown; pair of oblique brownish bands; mesonotum yellowish brown to brown; paired longitudinal brown bands run centrally along initial adult sutures and several spots of same colour centrally and laterally; metanotum brown, unclear brownish smudge centrally (Figure 2a and Figure 3a). Light brown to brown pleurites on lateral sides of thorax (Figure 2b and Figure 3b). Ventral side of thorax paler than dorsal side; sterna yellow to yellowish-brown. Legs pale. Femora and tibia yellowish brown to light brown; apex of tarsi and tarsal claws intensively brown (Figure 2b and Figure 3b). 

Abdominal terga (Figure 2a,b and Figure 3a,b) yellowish brown to brown. Oblique narrow brown spots anteriorly on tergum I, clearly visible on paler background; similar oblique spots on tergum II; combination of short oblique spots anteriorly, and two elongated spots centrally, on terga III and VI (occasionally V); color pattern on terga VI–IX is represented by a combination of triangular shaped spots anteriorly and two nearly rounded spots centrally; two elongated dark smudges centrally on tergum X. Abdominal sterna yellow to yellowish-brown, paler than terga; a pair of unclear smudges centrally on sterna II–VII (VIII). Cerci and paracercus pale, yellow to dirty yellow and yellowish-brown, and slightly darker distally. 

Hypodermal coloration. Hypoderm without contrasting markings.

**Head**. Solitary *B* and *Hr* setae and occasional *FT* setae on clypeus sparsely scattered across lateral side of frons. Frontal suture widely U-shaped (Figure 9d). Antennae distinctly longer than 1/2 of body length. Scape and pedicel with *FT* and *Hr*, and more abundant *B* setae located mainly along distal margin of each segment; few *Ca* setae along distal margin of pedicel; no peculiar cuticular corrugation (which is present in some representatives of the *Baetis alpinus* species group and in the closely related *B. lutheri* and *B. pavidus* species groups as defined by Bauernfeind and Soldán [4]). Distal margin of flagellar segments composed by large spines rounded apically, alternating with *FT* and occasional *TR* and *B* (Figure 9a–d, Table 2).

*Mouthparts*. Labrum moderately oblong-shaped, width/length ratio of labrum 1.40–1.45; strong posterior convergence of lateral margins; 1 + 14–18 long submarginal setae on dorsal surface arranged in a single irregular row; 6–12 smaller stout setae laterally on both margins; dorsal surface of labrum covered with densely arranged *B* and only a few *FT* and *Hr* setae grouped posterolaterally. Median incurvation of labrum anterior margin moderately shallow and wide (Figure 5a,b and Figure 9e).

Outer mandibular incisor group relatively broad, rounded at apex, moderately separated from inner incisor group by a shallow incision; inner incisor group not prominent, bearing 3–4 small teeth; right and left prostheca nearly of same size, asymmetrical, bearing 8–10 apical teeth (Figure 5d,e). 

Maxillary palp two-segmented; segment 1 larger than segment 2; segment 2 with almost parallel outer and inner margins, asymmetrical only apically; tip of segment 2 with a small conical protuberance bearing 2–6 stout setae; surface of both segments with *B* setae (same type of setae is described as uniporous sensillum basiconicum sensu Gaino and Rebora [30]: 449, Figures 19−21)), densely scattered on distal part of segment 2. Three enlarged, acute teeth situated apically on maxillary galea-lacinia, accompanied by one row of long setae (Figure 6a,b, Figure 9f and Figure 10a–c). 

Hypopharynx as in Figure 5c, with prominent central lobe in lingua and marked apical hump on superlinguae.

Labium with relatively slender glossae and paraglossae; glossae slightly shorter than paraglossae; up to 14 stout bristles along inner margin and up to 6 stout setae on outer margin of glossae; 10–14 long bristles grouped into two rows on tip of paraglossae; 6–10 long bristles along outer margin and 4–5 medium sized setae on ventral side of paraglossae. Segment 2 of labial palp 1.20–1.35× longer than segment 3, covered with sparse *B* and *Hr* setae and without considerably developed apico-internal lobe; segment 3 nearly symmetrical and evenly rounded apically, slightly broader than long (length/width ratio 0.95–1.10); 18–25 slender, pointed, stout setae on dorsal apical surface of segment 3, accompanied by long *Hr* and slightly shorter *B* setae; quotient *q* 1.00–1.08 (Figure 6d–i and Figure 10d).

**Thorax.** Surface of pronotum with a few *B* and occasional *FT* and *Hr* setae. Sternal protuberances on meso- and metathorax visible, pointed apically, yellowish-brown to brown. Outer margin of femora with 2–4 rows of long, tiny bristles proximally arranged in 1–2 rows, and one row of shorter bristles distally; long marginal bristles pointed and bluntly pointed apically, alternating with submarginal *STS-p* and *STS-bp* setae, elongated *Hr* and occasional *B* setae; inner margin with sparse row of small *STS*-*bp* setae; surface of femora with *STSs-bp* and *STSs-ov* setae and group of tiny setae (*Hr* and more abundant *FT*). Outer and inner margins of tibiae with small *STS-p* and *STS-bp* setae, alternating with *Hr* and *B*; stout setae more abundant along outer margin; surface of tibia with sparse small *STS-bp* and *STSs-ov* setae, more abundant along patella–tibial suture. Tarsi with 7–11 middle to elongated *STS-p* setae along inner margin, and several small to middle sized *STS-p* and *STS-bp* setae on outer margin; surface and both margins of tarsi covered with more abundant tiny *Hr* setae, occasional *FT,* and small *STS-bp* setae. Tarsal claws relatively short, moderately hooked; 8–11 teeth arranged in a single row along claw; two subapical hair-like setae (Figure 7a–c and Figure 12a–d).

**Abdomen.** Posterior margin of terga with broad triangular spines of different sizes, bluntly pointed or occasionally pointed apically, and occasionally a few small spines subapically; broader, nearly symmetrical spines along posterior margin of terga III–VIII (IX); marginal broad spines alternating with 1–3 tiny *FT* and isolated *Hr* setae. Surface of terga with a few moderately elongated, tongue-shaped [*SC-tg*] scales, mostly concentrated centrally near posterior margin of segments; solitary scale sockets concentrated also posteriocentrally, mainly on terga IV–VIII; solitary *Hr* and more abundant *FT* setae stretched over whole surface of terga I–X. Posterior margin and surface of sterna without spines, stout setae or scales, with *B* and *Hr* setae only (Figure 11a–d). 

Paraproct plate as in Figure 8c, relatively short, cercotractor not elongated; no prominent spines along inner margin of paraproct (only a few weakly separated small spines were poorly visualized); solitary *FT* and *Hr* along of inner margin; same type of setae sparsely scattered on paraproct surface distally; inner margin of cercotractor with inconspicuous/subtle range of bluntly pointed spines spread centrally, a few *FT* and *Hr* setae (Figure 8c). 

Tracheal gills white to light brown, with slightly pink center; no elongate gill plates; gills moderately asymmetrical, blunt apically, inner margin broadly rounded; gills II–VI asymmetrical; gills I and VII less asymmetrical; serrated margins of gills relatively well marked, with tiny *Hr* setae inserted at small, articulated bases; *FT* and *Hr* setae scattered on dorsal surface of gills; tracheation poorly visible (Figure 8a,b and Figure 11e,f).

Cerci 0.54–0.57× body length. Paracercus consists of more than 50 segments. Posterior margin of cercal and paracercal segments each with a row of broad, triangular spines, mainly blunt and bluntly pointed apically, alternating with *B* setae, occasionally grouped with 2–3 such types of setae (Figure 12e,f). 

Morphological characters to distinguish *B. dihyae* **sp. nov.** from other representatives of the *Baetis alpinus* species group, especially from closely related species, are given according to Müller-Liebenau [2], Thomas et al. [46], Thomas and Dia [47,48], Peru and Thomas [49], Jacob [3], Kluge and Novikova [50], Bauernfeind and Soldán [4], and Sroka et al. [45] and are summarized in Table 3. 

**Male and female adults**. Unknown.

**Distribution and biology.** *Baetis dihyae* **sp. nov.** is known so far only from two localities of Aurès streams (Figure 1). The species is probably a micro-endemic of the High Mountain ranges of this region. Based on present data, the new species seems to be rare, or localized to several water sources only, since it has not yet been collected from other localities, despite present records on mayfly fauna of the Aurès Mountains. 

**Figure 13:**
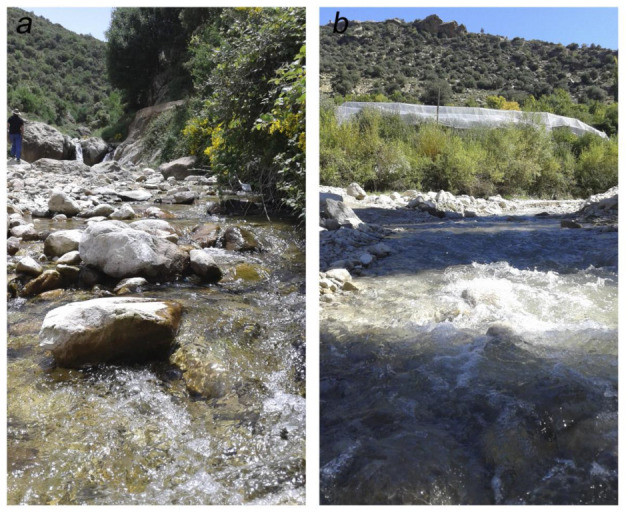
Sampling sites of *Baetis* (*Baetis*) *dihyae* **sp. nov.**: (**a**) Yabous (type locality), app. 1420 m a.s.l. and (**b**) Inoughissen stream (Wilaya de Khenchela), app. 1670 m a.s.l. (photos by B.M. Dambri).

Larvae of *B. dihyae* **sp. nov.** were found solely on stony substrates of different size in smaller streams about 1.50–3.50 m width (Figure 13a,b), with an average depth of 17–25 cm and a variable velocity of flow (0.5–1.0 m/s). The water temperature ranged from 5–19 °C during the year, the maximum occurring in August. Dissolved oxygen varied from 7.6–9.8 mg/l, oxygen saturation from 80–126%, and conductivity from 450–500 μS/cm; pH 7. The larvae of *B. dihyae* **sp. nov.** were collected with other Ephemeroptera: *B. sinespinosus*, *B. chelif,* and *Ecdyonurus aurasius* Dambri, Benhadji & Sartori, 2022; Trichoptera: Polycentropodidae *Plectrocnemia conspersa* (Curtis 1834), Psychomyiidae *Tinodes dives* (Pictet 1834), and Philopotamidae *Wormaldia variegata numidica* Vaillant, 1974 [51]; Diptera Simuliidae *Simulium* (*Eusimulium) velutinum* (Santos Abreu, 1922) [52], and Chironomidae: *Diamesa* (*Diamesa) insignipes* Kieffer 1908 and *Eukiefferiella mino*r (Edwards 1929) (B.M. Dambri, *unpublished data*).

### 3.2. Molecular Study

**Figure 14:**
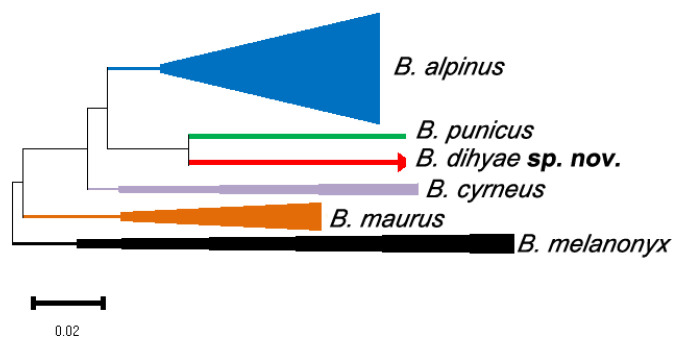
COI neighbor-joining tree, using Tamura–Nei model, with collapsed haplogroups of *B. alpinus* in blue, *B. punicus* in green, *Baetis dihyae* **sp. nov.** in red, *B. cyrneus* in purple, *B. maurus* in orange, and *B. melanonyx* in black.

**Figure 15:**
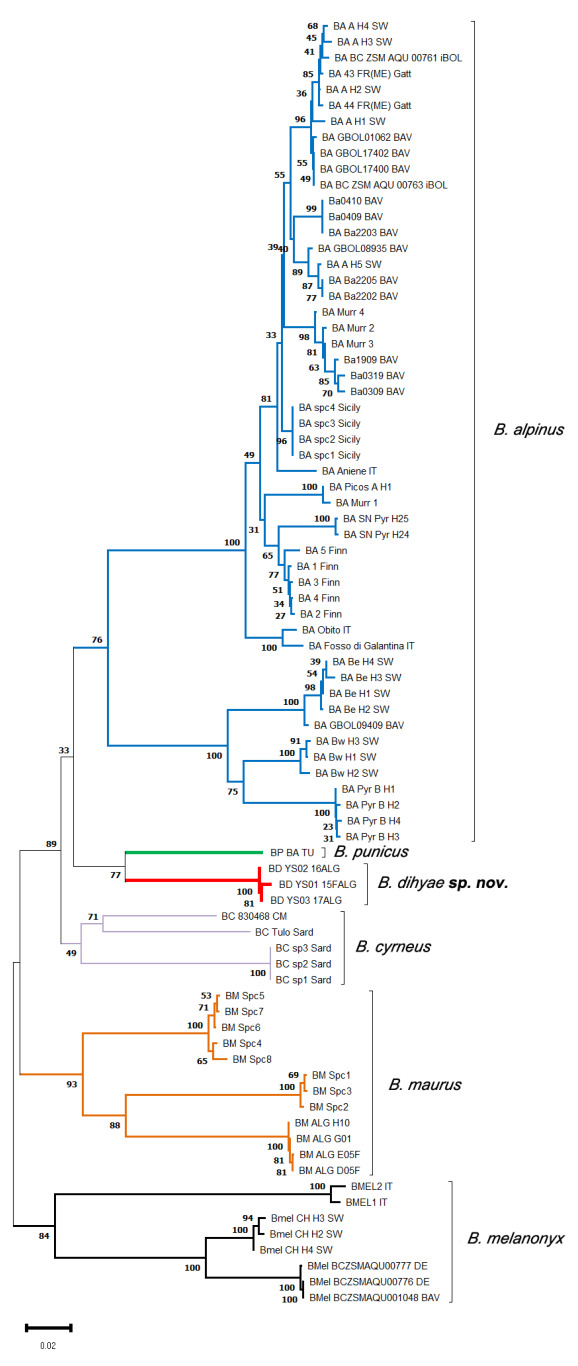
COI neighbor-joining tree with all haplogroups.

The neighbor-joining tree analysis helped in delineating six haplogroups of six *B. alpinus* species group species’ sequences (Figure 14 and Figure 15, Table 4). Three *B. dihyae* **sp. nov.** sequences formed a strongly supported monophyletic haplogroup (with a 100% bootstrap). This haplogroup is also supported (bootstrap = 77%) as the single-branched sister clade of one specimen’s sequence of *B. punicus* species from Tunisia. On the other hand, the interspecific genetic distance between *B. dihyae* **sp. nov.** and *B. punicus* haplogroups is quite high (12%), and the latter also morphologically discriminated from its congener above. The *B. dihyae* **sp. nov.** haplogroup is highly divergent genetically from all the remaining haplogroups (from 19–25%).

## 4. Discussion

### 4.1. Affinities

Formerly, Thomas and Dia [47] identified a new subgenus, *Patites* Thomas & Dia, 1999, for two species included in the *Baetis alpinus* species group sensu Müller-Liebenau [2], namely *B. melanonyx* (Pictet, 1843) known throughout Europe, *B. baroukianus* Thomas & Dia, 1984 described from Lebanon, and related species. Afterwards, Godunko et al. [5], whilst describing a new species *B. cypronyx* from Cyprus, indicated that it is closely related to *B. melanonyx* and *B. baroukianus*. However, as a precaution, the attribution of *B. cypronyx* to the subgenus *Patites* was not considered by these authors, as this group lacks global revision (J-L. Gattolliat, *personal communication*), and only 13 species were reassigned to this subgenus by N.J. Kluge (see web resource Ephemeroptera of the World [53]). Most recently, *Ephemeroptera of the World* [53] implemented the subgenus *Patites* for all West Palearctic species, and it was also used by Gattolliat et al. [54] for the identification key of Maghrebian Baetidae larvae. Moreover, this key also aligned with distinguishing characters proposed by Thomas and Dia [48], namely the rectangular labrum with one row of fringed distal margin bristles; fused, triangular-shaped mandibular canines; pointed spines on the posterior margin of tergum IV; and a reduced paracercus.

Bauernfeind and Soldán [4] and Godunko et al. [5] avoided this taxonomic concept and adopted the *Baetis alpinus* species group, one of the groups proposed by Müller-Liebenau [2] and endorsed by Jacob [3]. It was again shown that the delimitation of the species of the *Baetis alpinus* species group can be rather difficult and unclear because there are high levels of clinal variability of taxa included, combined with the disjunctive area of many species (for more details see Godunko et al. [5]). 

Additionally, it should be noted that according to the larval characters proposed earlier for the establishment of *Patites*, *B. dihyae* **sp. nov.** undoubtedly does not belong to this subgenus, being a representative of a different and more numerous species group—*Baetis* gr. *alpinus*. Furthermore, almost all the features proposed for *Patites* are not attributed only to this subgenus as they are shared by other representatives of the *Baetis alpinus* species group:

**(i)**  Labrum: the rectangle-shaped labrum attributed to Patites is as well observed in new species *B. alpinus*, *B. nubecularis,* and *B. pasquetorum*. The long, submarginal bristles on the dorsal surface of the labrum are disposed in one row (irregular in *B. dihyae* **sp. nov.**), and a mean number of bristles ranging between 1 + 14–21 is also a characteristic of whole *Baetis* gr. *alpinus*, which is characterized by the presence of 1 + 10–22 to 1 + 18–22 such bristles (e.g., *B. alpinus*);
**(ii)**  Maxillary palp: *B. dihyae* **sp. nov.** bears more than one stout seta on the tip of the maxillary palp, unlike the subgenus *Patites*;
**(iii)**  Mandibles: the prominent mandibular incisors are described as triangular and often fused for all representatives of *Patites* and the *Baetis alpinus* species group;
**(iv)**  Labial palps: segment 3 is listed as stout in *Patites*; for *B. dihyae* **sp. nov.** and closely related species (see Table 3), it is more or less rounded and symmetrical as in, e.g., *B. alpinus*;
**(v)**  Posterior margin of abdominal terga: a row of strong spines is characteristic of all congeners of the *Baetis alpinus* species group sensu Müller-Liebenau [2] and is listed in Bauernfeind and Soldán [4];
**(vi)**  Surface of abdominal terga: in contrast to *Patites* with numerous scales and their bases densely scattered on the surfaces of terga; *B. dihyae* **sp. nov.** is characterized by a small number of such scales along the anterior margin of the segment and their absence (including scale bases) anteriorly and centrally;
**(vii)**  Paracercus: this character excludes *B. dihyae* **sp. nov.** from the subgenus *Patites*, which comprises species with a diminished paracercus; *B. dihyae* **sp. nov.** has a long paracercus as in, e.g., *B. alpinus*.

Together with *B. alpinus*, *B. nubecularis*, and *B. pasquetorum*, *B. dihyae* **sp. nov.** occupies an independent position within the *Baetis alpinus* species group due to features in the (**i**) structure of the tip of segment 2 of the maxillary palp, which bears more than one stout setae, and (**ii**) a well-developed paracercus as long as approximately 0.5 of the cerci length (see Table 3). While *B. nicolae* Thomas & Gazagner, 1983 and *B. punicus* Thomas, Boumaiza & Soldán, 1983 are both characterized by apical setation of the maxillary palps with one to four (rarely a fifth) setae of different sizes, the paracercus is always diminished to a few segments in these species, in contrast to *B. dihyae* **sp. nov.**

*Baetis punicus* seems to be the closest genetically to *B. dihyae* **sp. nov.,** with well supported sister-clade haplogroups in the COI tree; yet, with significant genetic distance, these two species share indeed few features: scape and pedicel without stout setae, surface of tegites covered by semi-lunar impressions, and well-developed gills with gill IV and gill V lengths equal to the length of two terga joined together (Thomas et al. [46]). However, they are clearly different by the following characteristics: the shape of the labrum and mandibles, as *B. punicus* labrum and mandibles (fused with inner inciser group), are similar to those of *B. alpinus* (Table 3); the surface of terga as *B. dihyae* **sp. nov.** bears some tongue-shaped setae *SC-tg*; the length and number of segments of the paracercus are excessively longer in *B. dihyae* **sp. nov.** and of up to 60 segments, while it is shorter in *B. punicus,* possessing only up to 25 segments.

Thus, biomolecular investigations of more populations of *B. dihyae* **sp. nov.** and *B. punicus* are strongly needed to explore this monophyly as the DNA barcoding can show some limitations in species haplogroup delineation (e.g., Funk and Omland [55]; Duran et al. [56]), especially within small sample sizes sequences.

Some clear differences between the new species and taxa listed above are recognized in the structure of the mouthparts. The labrum of a new species is rectangular-shaped; however, the lateral posterior margins are more convergent than in the closely related congeners discussed above. The shape of labial segments 2 and 3 of *B. dihyae* **sp. nov.** slightly differs from *B. alpinus*, *B. nubecularis,* and *B. pasquetorum*. Nevertheless, the new species, together with *B. alpinus,* is characterized by the presence of numerous stout setae situated dorsally on the tip of labial segment 3 (Table 3). Some minor differences, especially in contrast to *B. nubecularis* and *B. pasquetorum,* can be observed in the number of long submarginal and short marginal setations of the labrum and the shape of prostheca setation. 

Well-defined differences between the species discussed can be found in the shape and other characteristics of the body setation. In contrast to *B. nubecularis* and *B. pasquetorum*, *B. dihyae* **sp. nov.** possesses weakly developed setation of the abdominal terga surface, with few tongue-shaped scales grouped mainly along the anterior margin of segments. The same type of scales is scattered on the whole surface of abdominal terga in *B. alpinus* [2,57] and absent in *B. nubecularis* and *B. pasquetorum*. The same is recognized in the case of the scale sockets, which are present in the new species and *B. alpinus* only. Additionally, in contrast to all these species, *B. dihyae* **sp. nov.** can be separated by the presence of an irregular submarginal row of small setae, well recognizable on abdominal terga III–VIII. Finally, the paraproct plate of *B. dihyae* **sp. nov.** characteristically lacks stout setae in contrast to other species that have a few bluntly pointed submarginal setae; the inner margin of the paraproct in *B. dihyae* **sp. nov.** occasionally bears one incospicuous spine, markedly contrasting with developed setation consisting of 4–15 spines in *B. alpinus*, *B. nubecularis,* and *B. pasquetorum* (Table 3).

### 4.2. Biogeographical Considerations

#### 4.2.1. Overview of *Baetis alpinus* Species Group in the West Palearctic

**Figure 16:**
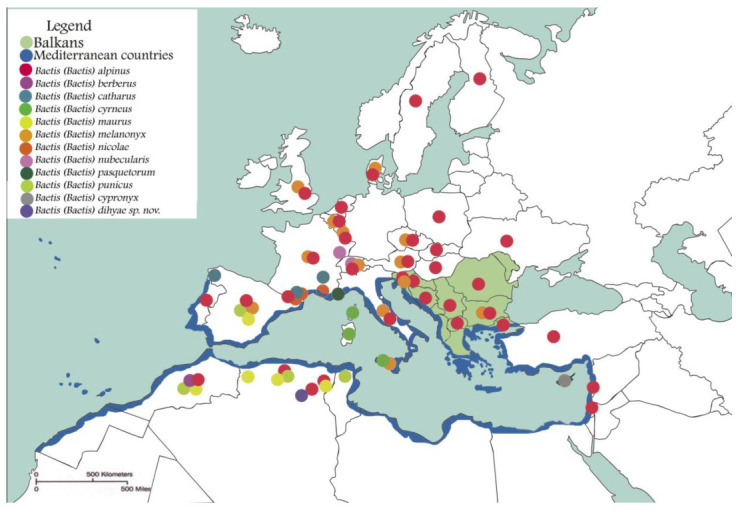
Diversity and distribution of the *Baetis alpinus* species group in the Western Palearctic.

Primarily, the *Baetis alpinus* species group, altogether, is known to encompass cold stenothermic elements [4] and is somewhat restricted to higher altitudes and mountainous streams (200–2600 m a.s.l) ([4,35,39,58,59,60,61]. They occur in humid to sub-arid climates in intermittent or permanent streams in the West Palaearctic realm, from the Alpine streams in Europe down to the Atlas Mountain ranges in the Maghreb; they are also reported from the Mediterranean islands, e.g., Sardinia [62] and Cyprus [5]. Amongst the ten countries of the Balkan Peninsula, *B. alpinus* is spatially dominant, and *B. melanonyx* is reported from Slovenia, Albania, and Bulgaria only. The questionable record of *B. melanonyx* from North Macedonia in Memeti et al. [63] needs to be confirmed. Latitudinally, they are distributed from ca. 50–55° northern latitude to the Mediterranean and North Africa (the southern limit is at 35°16′42″ N, 6°32′34″ E at 1670 m a.s.l. in the Inoughissen site, Wilaya de Batna, Algeria, for *B. dihyae* **sp. nov.**). Their eastern limit is in the Levant [4] and the western one is in the Ibero-Moroccan region (Figure 16).

Around the Mediterranean basin, several endemic species of the *Baetis alpinus* species group appear: *B. pasquetorum* in the Southern Alps of France, *B. cyrneus* in Corsica, *B. cypronyx* in Cyprus, and *B. maurus* with apparently different species populations in Spain and in North Africa [9]. Additionally, two other species are known from the Maghrebian area, namely *B. berberus* Thomas, 1986, considered as a Moroccan endemic, and *B. punicus*, described from Tunisia and occurring in the whole Maghreb [4]. 

Considering only the *Baetis alpinus* species group in North Africa, records exist from Morocco and Northeastern Algeria [10,11,12] and in this present study. However, several studies containing molecular analyses of studied taxa have unveiled the rich diversity of the *Baetis alpinus* species group and nominal species *B. alpinus*, with several cryptic or sibling-species isolated in the West Palearctic [6,7,9,10,11,12,38,54,64]. Thus, the reliable number of taxa of mentioned species group within the Mediterranean region and North Africa should be markedly rich and diverse. 

In the Maghreb, recent studies on the taxonomy and distribution of Ephemeroptera, supported by molecular analyses, argue that the Maghrebian *B. alpinus* represents a cryptic complex of the species in Morocco (morphological studies still not published), where specimens have been shown to be genetically distant from Iberian populations [10] and of the putative endemic in Algeria [11,12,54]. These populations occur 20–1500 m a.s.l. in northern Morocco [10]. In northeastern Algeria, they appear at the highest site reported in Samraoui et al. [11] (see site O. Nil, 864 m a.s.l), when in the Kebir-East, material was collected at ca. 1200 m a.s.l. in the mountains of Mount Ghora, Kroumiria [12], and have reached peaks of 1420 m in the Yabous site and 1670 m in the Inoughissen site (see present study). 

In the Kebir-East, which is the second largest catchment in northeast Algeria, the populations of *B. alpinus* species group are widespread together with *B. atlanticus* Soldán & Godunko, 2006 and *B.* cf. *maurus* [12]. However, they are rare in the Seybouse catchment (the first and largest catchment in northeast Algeria) [11] and are accompanied by the larvae of *B. maurus* and rarely with taxa belonging to the subgenus *Rhodobaetis*.

The *Baetis alpinus* species group has apparently never been collected from the Tafna basin (northwest Algeria), although it has been investigated. Only *B. maurus* is found in the Tafna basin, along with quite abundant representatives of *Rhodobaetis*, namely *B. sinespinosus* and the scarce *B. atlanticus* [9,65,66,67]. The absence of *B.* cf. *alpinus* from this river-basin is quite surprising, as the Tafna watershed is juxtaposed with the Oriental Moroccan region, where it is reported [10]. On the other hand, *Baetis* cf. *alpinus* elements are also believed to occur in central Algeria (Kabylie), but no published data are available. This region is one of the most diversified in Algeria [24,26], possibly correlated to the high altitude streams and profuse water flow. 

Farther East in Tunisia, Maghrebian representatives of *B.* cf. *alpinus* have never been recorded, and only *B. punicus* from the same species group was recorded [54,68].

#### 4.2.2. On the Origin of *Baetis* Leach, 1815 in North Africa

According to Razeng et al. [69], speciation in mayflies has been associated with fluctuations in sea levels, continental uplift, and glaciation as a gene flow barrier (see also Bisconti et al., Sekiné et al., Theissinger et al. [70,71,72]). Indeed, several geological and climatic event developments may have shaped the mosaic pathway of mayfly distribution in the Mediterranean basin and the Maghreb area in particular. The geological pattern of the Mediterranean was mainly modelled by the Alps orogenesis that started in the Cenozoic, from the Oligocene to the late Pliocene [73,74]; it provoked major changes in the adjacent area between Europe and Africa due to extensional tectonics. 

There are few palaeozoogeographical investigations involving aquatic insects of Pterygota in the North African or Mediterranean region (for Odonata see [75]). Except for some species, mayflies are commonly considered poor dispersers, exhibiting a reduced gene flow between catchments, thus promoting genetic divergence [69]. 

However, the opposite has been demonstrated. Namely, the mayfly taxa successfully crossed from islands to continent and vice versa, as shown, e.g., for Madagascar and Africa [76], the Iberian Peninsula and Macaronesian islands [41], and for latter and North Africa [9]. Thus, Ephemeroptera unite all the three qualities to test biogeographical patterns recommended by Schmitt [77], especially (i) high dispersal ability, and then, (ii) once established, large stable populations (iii) that exhibit more or less sedentary and non-migratory behaviour. Generally, the Maghreb populations of the three countries show discrepancies in species richness, with west–east decrease. However, such differences in the abundance and population structure are not a unique situation, and east–west differentiations were also noticed in reptiles [78,79,80] or mammals [81] due to physical barriers such as the Atlas Mountains. 

Forty years before, Soldán and Thomas [24] acknowledged the relationships between North African Ephemeroptera and European fauna and almost excluded any connection between West Palearctic and Afrotropic (or Ethiopian) fauna. Later, Jacob [82] affirmed that the North African Mediterranean is part of the Palearctic, as it is evidently similar to the southern European one at all taxonomic levels (although not as diversified species-wise) and shares the same Laurasian origin. Nowadays, only a few European species are confirmed, such as *Habrophlebia fusca* (Curtis, 1834) (Leptophlebiidae) *Paraleptophlebia cincta* (Retzius, 1783), *Potamanthus luteus* (Linnaeus, 1767) (Potamanthidae), *Ephemera glaucops* (Pictet, 1843) (Ephemeridae), *Ephoron virgo* (Olivier, 1791) (Polymitarcyidae), *Serratella ignita* (Poda, 1761) (Ephemerellidae), *Caenis luctuosa* (Burmeister, 1839) (Caenidae), and *Caenis pusilla* Navás, 1913) [26]. Many other species have been assigned as local endemics [54]. 

Recently, it has been increasingly argued that during the transit from southern Europe through the Mediterranean islands to North Africa, populations of several species groups within the genus *Baetis* have acquired distinct new morphological characteristics and molecular identities—adaptations that have raised them to the rank of a distinct new species with microendemic status [9,54]. 

Following a north–south gradient (Europe to North Africa), the track of the widely spread European species *Baetis* (*Rhodobaetis*) *rhodani* (Picteti, 1843) is lost, and no record is observed from the Macaronesian Islands (Table 5). From Madeira, the record of *B. rhodani* is based on misidentification (see Bauernfeind and Soldán [4]) and represents the Altlanto-Mediterranean *B. atlanticus*. This latter species is encountered further in the Maghreb, joining two endemics from the subgenus *Rhodobaetis*, namely *B. chelif* and *B. sinespinosus*. The species *B. enigmaticus* Gattolliat & Sartori, 2008 is endemic of Madeira. Further South in Macaronesia, *B. canariensis* Müller-Liebenau, 1971; *B. enigmaticus* Gattolliat & Sartori, 2008; *B. nigrescens* Navás, 1931; and *B. pseudorhodani* Müller-Liebenau, 1971 characterize the Canary Islands [83,84,85,86,87,88] (Table 5). Due to past misidentifications and the cryptic character of many *Baetis* and *Rhodobaetis* representatives [2,86,89,90,91,92,93], it is only recently that the taxonomic history of the *Baetis* species in the Macaronesian Islands has been substantially settled [83,84,85,94]. Gattolliat et al. [94] unveiled the high endemism of the *Baetis* species in the Canary Islands, with species solely bound to a unique island (Table 5).

Concerning the Afrotropical mayflies, a Gondwanan origin is likely. They could not have crossed the Sahara Desert belt, even with the presence of the Nile as a semi-transcontinental river, flowing from Lake Victoria (Uganda, Kenya, Tanzania) to the Nile delta (Egypt). This is probably due to the lentic/potamophilic character of the Nile River, which starts from Sudan’s hot and desert climate in the Sahel and Sahara of Northeastern Africa—not a habitat favoured by rheophilic or cold stenothermic mayflies. 

To understand the migration routes within the region discussed, paleogeography should be considered. Major events occurred mainly between the Late Oligocene to the Quaternary, with the most prominent shifts happening during the Miocene, Pliocene, and Pleistocene series (e.g., Messinian Salinity Crisis (MSC); Quaternary ice age and deglaciations, Alps orogenesis) [73,74]. Also, during Quaternary glaciation/deglaciation, the Saharan belt moved vertically back and forth many times, resulting in differentiation of some terrestrial populations of Northwest and Northeast Africa, such as beetles, snails, reptiles, and mammals such as shrews [78,80,81,95,96,97]. 

However, the Saharan Belt was not an obstacle for some Pterygota, such as the order Odonata (another group in the Paleoptera), as they are strong fliers compared to Ephemeroptera. Thus, approximately 20 Afrotropical species of Odonata reached North Africa, and even further North to Europe, the Levant, and even the Arabic or Persian Gulf, through the Nile Corridor, which seemingly acted as a highway for most of these species; six of these species were found only in Egypt [75]. In the previously cited study, only 2 species (belonging to the Coenagrionidae family) of the 19 species are reported from the Levant (*Pseudagrion torridum* Selys, 1876) and the Arabic Gulf in West Saudi Arabia (*Ceriagrion glabrum* (Burmeister, 1839)), and 11 of the 19 species are or were (and now extinct) found in Algeria: it took only a few decades for these species to spread. The Libellulidae *Trithemis annulata* (Beauvois, 1807) even crossed to the Canary Islands due to recent climate change and was considered by Boudot et al. [75] as an indicator of expansion. 

Thus, the humid periods in the Pleistocene or Sahelian Holocene shifted the Sahara belt and so facilitated faunal movement northward, also explaining the presence of relict Afrotropical species in the Maghreb region. Nevertheless, after these periods, these regions most likely represented a barrier to the expansion of Afrotropical taxa of aquatic fauna, as was shown in the case of Odonata [75]. Another Afrotropical colonizer is the biting midge *Culicoides imicola* Kieffer, 1913 (Ceratopogonidae), which formerly had an Afrotropical distribution that might have extended from the West Palearctic into the Mediterranean basin due to climate change in the Late Pleistocene or Early Holocene [98].

#### 4.2.3. Exploration of Possible Colonization Pathways

**Figure 17:**
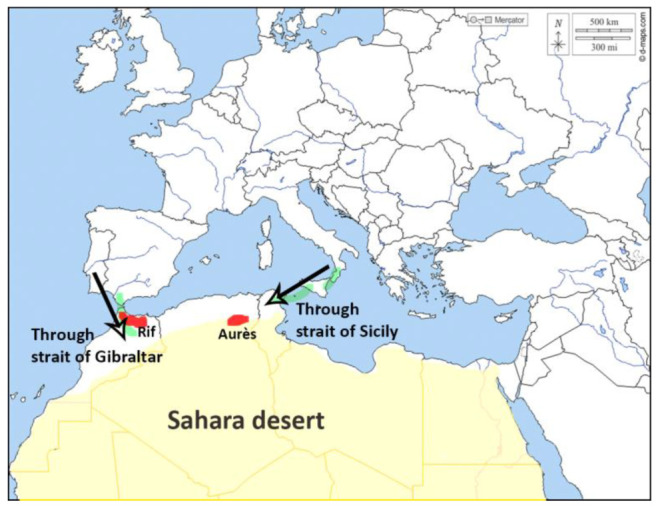
Colonization pathways of the *Baetis alpinus* species group in North Africa (for detailed description, see Section 4.2.3). Green areas represents the connection of land-bridges (strait of Gilbraltar and strait of sicily) to North africa. Black arrows represent the sens of colonization.

Through examining the history of fauna, whether of vertebrates or invertebrates, and the recurrent and consistent findings of colonization patterns, the movement of *Baetis* representatives between Europe, Northwest Africa, and subsequently, Algeria, can be understood, with the land-bridges ‘the Strait of Gibraltar’ and ‘the Strait of Sicily’ acting as colonization routes (see Figure 17) as follows:

- Eastern Algeria colonization path (Strait of Sicily):

Schmitt [77] considered the Adriatic–Mediterranean refugial area (continental Italy) to be geographically less diversified and smaller and with generally less complicated paleogeographical processes than the Iberian Peninsula and the Atlantic–Mediterranean region. Even if the Apennines were supposedly connected to Tunisia by Sicily and Malta since the Oligocene (30 Ma) [73,74], this colonization path does not seem to be likely, at least not for all the majority of mayflies as their diversity is seemingly much higher in the western part of the Maghreb than the eastern part, with Ibero-Maghrebian closely related representatives differing morphologically, genetically, or both (e.g., see Gattolliat et al. [54] for some Baetidae). However, Pliocene sea-level changes could have had unanticipated effects on North African freshwater taxa.

While the data about aquatic insects are scarce, some information on other groups of aquatic biota were published. For example, Carranza and Wade [99] speculated that ancestral populations of *Pleurodeles* Michahelles, 1830 (Amphibia: Salamdridae) found themselves isolated in the Edough Peninsula (northeast Algeria) after the division of the Afro-European land-bridge (ca. 5.3 Ma ago), and that marine transgression during the Pliocene might have transformed Edough Peninsula into an island, initiating speciation (ca. 4.2 Ma ago, during the Upper-middle Pliocene). This speciation resulted in two new species restricted to the ‘fossil island’ Edough Peninsula, separated by lowland marshes from the rest of the continental land, and one other species inhabiting the rest of Algeria and Tunisia [99]. The reduced diversification of mayfly fauna of North Tunisia compared to Algeria and Morocco can be explained the same way, as well as the isolation of the Sardinia vagrant *Habrophlebia consiglioi* Biancheri, 1959 (Leptophlebiidae) in northern Tunisia [100]. The latter probably had a successful colonization between the MSC (6 Ma) and the Pliocene (ca. 5 Ma). 

In many palaeozoogeographical studies, the evolution of circum-Mediterranean Arthropoda species was shown to be crafted by paleoclimatic and geologic events in several ways. In a climate model, Trájer et al. [101] implied that the desiccation of the Mediterranean Sea during the Messinian (end of the Miocene) was a turning point for three Mediterranean sandfly species of the genus *Phlebotomus* (Psychodidae): speciation was rapid, but migration was not. According to the same authors, the intense hot climate and the high salinity of the Mediterranean abyssal plain during this time inhibited Europe–North Africa cross-migration, whether through the Gibraltar or Sicily straits. On the other hand, several former studies qualified the MSC as ‘the trans-Mediterranean migration period’ of sandfly ancestors, and thus, the Sicilian Strait route should not necessarily be disregarded. Several studies also addressed the possibility of faunal north–south (Italian Peninsula–North Africa) exchanges of land biota and freshwater invertebrates [101,102,103].

- Western Algeria colonization path (Strait of Gibraltar):

The West Mediterranean area (Atlantic–Mediterranean refugial area sensu Schmitt [77]), including the Iberian Peninsula and Maghreb, is known as the most studied shelter for Southern species during the MSC and the glaciations cycles (see also Pinto-Juma et al. [97]).

The Strait of Gibraltar opening and separation of North Africa (end of the MSC; ca. 5.33 Ma), followed by the historically unequalled decrease in sea levels during the Late Glacial Maximum, unveiled continental shelves on both sides of the Mediterranean and new islands [104]. 

This acted as a “stepping stone” to the flora and fauna from both continents [97]. These processes divided the Atlantic–Mediterranean refugial area and enhanced the possibility of radiation by vicariance for various taxa due to isolations [77,97,105,106,107]. 

For the mayflies, the West Mediterranean north–south affinities of *Baetis* suggest a rather western Mediterranean dispersal or migration pathway [9,54]. 

Based on this suggestion, we could suppose that colonization of North Africa by *Baetis* ancestors probably began after the Middle Miocene (15 Ma) and not before. Before the Oligocene, there was more or less a stable climate and geological situation in Europe, and so *Baetis* fauna were not pressured to migrate and find new favourable ecological niches further in the south. 

In regard to the first Northwest African refuge (north of Morocco) and the expansion from the western to eastern Maghreb, the movement of fauna may have occurred through the Mauretanic Mediterranean, as suggested by de Lattin [108] based on butterfly studies—considering the fact that the Rif mountains more or less blocked the dispersal of many taxa previously cited. In the case of Central Europe, Monaghan et al. [64] suggested that the fragmentation of alpine streams by lakes or dams creates barriers that hinder the gene flow of *B. alpinus* and their habitat distribution along the streams and thus isolates populations. Furthermore, Leys et al. [6] highlighted a genetic divergence within cryptic lineages of *B. alpinus* in European mountain ranges and attributed this to spatial isolation among mountain massifs. Following the scheme of Leys et al. [6], geographic barriers and allopatric speciation induced by stream fragmentations are most likely also the reason behind the presence of several sibling species of the *Baetis alpinus* species group in the Maghreb, and due to the physical barriers of high mountain ranges, especially in Morocco and Algeria, in these countries the species can be found only in the north central and eastern regions. 

The hypothesis discussed above corroborates with Hrivniak et al. [109], which proved that the grouping within *Epeorus* (*Caucasiron*) Eaton 1881 species, in the Greater Caucasus range, happened between the Later Miocene and the Pliocene (between 10–5 Ma). Additionally, Poulakakis [110] described the MSC as one of the most pertinent geological events in the biogeography and biodiversity of both terrestrial and aquatic Mediterranean-dwelling fauna. Recently, Trájer et al. [100] proved that the MSC encouraged migration of some other sandfly species of the genus *Phlebotomus*. 

Northwest Africa is typically considered as the southern retreat for Northwest Mediterranean fauna during harsh climate fluctuations (e.g., Glaciations cycles) [97]. It is also considered that the land bridges of Gibraltar of Sicily acted as colonization channels during sea-level oscillations, and that the Strait of Gibraltar worked as a path of migration to the south (Maghreb) and of re-migration to the north (Iberian Peninsula) after better climate conditions returned [111,112]. 

Pinto-Juma et al. [97] demonstrated that Moroccan *Cicada* (Cicadidae) populations exhibit a greatly diversified haplotype diversity, which infers that they are demographically more stable in the Northwest African refuge than the Iberian populations. This can only confirm the hospitality of this refuge. Regarding mayfly populations of either side of the Rif Mountains, they are highly endemic, certainly due to the long height isolation period of their ancestor during the Holarctic. Finally, the checklist of Thomas [26] suggests that mayfly fauna of Baetidae and *Baetis* elements in North Central Algeria might be richer that the rest of the country due to high peaks and wadis with permanent and high discharge.

## 5. Conclusions

The purpose of this paper is to provide a comprehensive contribution to the taxonomy, diversity, and biogeography of the enigmatic *Baetis alpinus* species group with the description of a new species, *Baetis dihyae* **sp. nov.** This latter is differentiated at the nymphal stage from other representatives of the same species group, and the morphological affinities are closer to two Alpine endemic species (*B. nubecularis* and *B. pasquetorum*) when biomolecular ones show closeness to *B. punicus*. The new species is found to be part of a complex distribution of *Baetis alpinus* species group in the West Palearctic where we tried to understand the colonization history of the *Baetis* elements in the southern part of the Mediterranean basin with a special emphasis on the Maghreb region. The strongest paleozoogeographical hypotheses infer the Straits of Gibraltar and Sicily as favorite routes. However, in order to efficiently reconstruct the distribution and origin of the baetids *Baetis*, particularly the *Baetis alpinus* species group within the Maghreb and the circum-Mediterranean area, a much larger research effort is needed in collecting biomolecular and morphological data from different regions of Europe and North Africa in order to have substantial evidence for evolutionary relationships and colonization models.

## Figures and Tables

**Table 1:** COI sequenced specimens of *Baetis* (*Baetis*) *dihyae* **sp. nov.** with Genbank accession numbers and corresponding GenSeq nomenclature.

Species	Voucher Catalogue Number	Locality and Collector	Date	GPS Coordinates	GenBank ID	GenSeq Nomenclature
*Baetis dihyae* **sp. nov.**	BD_YS01_15FALG	Algeria, Khenchela Province, Yabous stream, Dambri B.M. leg.	28.X.2021	35°21′11″ N, 6°38′35″ E	OR644547	genseq-2 COI
*Baetis dihyae* **sp. nov.**	BD_YS02_16ALG	Algeria, Khenchela Province, Yabous stream, Dambri B.M. leg.	28.X.2021	35°21′11″ N, 6°38′35″ E	OR643835	genseq-2 COI
*Baetis dihyae* **sp. nov.**	BD_YS03_17ALG	Algeria, Khenchela Province, Yabous stream, Dambri B.M. leg.	28.X.2021	35°21′11″ N, 6°38′35″ E	OR643836	genseq-2 COI

**Table 4:** Genetic distances within (in blue cells) and between *B. dihyae* **sp. nov.**, *B. punicus*, *B. cyrneus*, *B. maurus*, *B. alpinus* and *B. melanonyx* haplogroups. Cells in blue background represent intraspecific distances.

	*B*. *dihyae* sp. nov.	*B. punicus*	*B. cyrneus*	*B. maurus*	*B. alpinus*	*B. melanonyx*
***B*** **. *dihyae* sp. nov.**	0.00					
***B. punicus***	0.12	n/c				
***B. cyrneus***	0.19	0.15	0.10			
***B. maurus***	0.23	0.22	0.22	0.11		
***B. alpinus***	0.20	0.20	0.20	0.24	0.09	
***B. melanonyx***	0.25	0.22	0.23	0.24	0.26	0.12

**Table 5:** *Baetis* Leach, 1815 representatives in the Macaronesian Islands.

Species/Islands	Azores	Madeira	Canary Islands	Cape Verde	Distribution
*Baetis* (*Rhodobaetis*) *rhodani* (Pictet, 1843)	All *Baetis* Leach, 1815 absent ^4^	Absent ^3,7^ (misidentification) teste ^4,5,6,10,12^ (misidentification) = *B. maderensis* (Hagen, 1865)	Absent ^12^ (misidentification) teste ^6,10,12^	Unknown ^12^	European
*Baetis* (*Rhodobaetis*) *atlanticus* Soldán & Godunko, 2006	–	Present ^12^	Absent ^12^	–	Atlanto–Mediterranean
*Baetis* (*Rhodobaetis*) *pseudorhodani* Müller-liebenau, 1971	–	Absent ^12^ (misidentification) teste ^13,14^ (misidentification) as *B. atlanticus* by ^12^	Present ^1,2,11^	–	Endemic
*Baetis* (*Rhodobaetis*) *enigmaticus* Gattolliat & Sartori, 2008	–	Present ^9^	Present ^9^	–	Endemic
*Baetis* (*Rhodobaetis*) *canariensis* Müller-liebenau, 1971	–	Absent	Present ^1,2,11^	–	Endemic of Gran Canaria
*Baetis* (*Rhodobaetis*) *gomerensis* Gattolliat and Sartori, 2018	–	Absent	Present ^8^	–	Endemic of La Gomera
*Baetis* (*Rhodobaetis*) *palmensis* Gattolliat and Sartori, 2018	–	Absent	Present ^8^	–	Endemic of La Palma
*Baetis* (*Rhodobaetis*) *tenerifensis* Gattolliat and Sartori, 2018	–	Absent	Present ^8^	–	Endemic of Tenerife
*Baetis* (*Baetis*) *nigrescens* Navás, 1931	–	Absent	Present ^1,2,11^	–	Endemic

Remarks: ^1^ [87]; ^2^ [86]; ^3^ [4]; ^4^ [89]; ^5^ [88]; ^6^ [92]; ^7^ [84]; ^8^ [93]; ^9^ [83]; ^10^ [2]; ^11^ [85]; ^12^ [82]; ^13^ [91]; ^14^ [90].

## Data Availability

All data are available in the paper. Requests for access to the material should be addressed to BD and RJG. This work has been registered online at Zoobank.org under LSID urn:lsid:zoobank.org:pub:2C4203AC-441C-4148-82DB-24F5C588D805. A new species is registered at Zoobank.org.

## Supplementary Materials

The following supporting information can be downloaded at: https://www.mdpi.com/article/10.3390/insects14110899/s1, Alignment S1: Alignment of nucleotide sequences of *Baetis alpinus* species group.
